# Relationship of Mitochondrial DNA Oxidation and Content with Metabolic Syndrome and Cardiovascular Risk in Obesity Phenotypes

**DOI:** 10.1155/2024/3008093

**Published:** 2024-09-11

**Authors:** Mailén Rojo, Hernán Pérez, Andrea Liliana Millán, María Constanza Pautasso, Gustavo Daniel Frechtel, Gloria Edith Cerrone

**Affiliations:** ^1^ Universidad de Buenos Aires Facultad de Farmacia y Bioquímica Departamento de Microbiología, Inmunología, Biotecnología y Genética, Buenos Aires, Argentina; ^2^ Universidad de Buenos Aires—CONICET Instituto de Inmunología Genética y Metabolismo (INIGEM), Buenos Aires, Argentina; ^3^ Servicio de Nutrición—Hospital de Clínicas José de San Martin, Buenos Aires, Argentina; ^4^ Fundación Héctor Alejandro (H.A) Barceló Instituto Universitario de Ciencias de la Salud, Buenos Aires, Argentina

## Abstract

**Objective:**

Obesity, chronic inflammation, and oxidative stress can influence mitochondrial DNA (mtDNA) content. Our objective was to evaluate the oxidation level and content of mtDNA and its relationship with metabolic parameters in metabolically healthy obese (MHO) compared to metabolically unhealthy obese (MUO) and normal weight (NW) controls.

**Materials and Methods:**

We studied 94 NW, 95 MHO, and 97 MUO individuals between 18 and 80 years old. Relative mtDNA content and mtDNA oxidation level (8-oxoguanine, 8-OxoG) were determined in peripheral blood leukocytes by the SYBR Green method of real-time PCR. One-way ANOVA and Tukey test were used to compare biochemical, clinical, and anthropometric characteristics, as well as mtDNA content and 8-OxoG.

**Results:**

A progressive decrease in mtDNA content was observed between NW, MHO, and MUO with significant differences in MUO vs. NW (*p*: 0.04). An increase in 8-OxoG was observed in MUO patients compared to the other groups (MUO vs. MHO *p*: 0.01; MUO vs. NW *p*: 0.04). mtDNA content was directly correlated with HDL-c (*p* < 0.01) and inversely with waist circumference (*p*: 0.01) and LDL-c (*p*: 0.05). mtDNA content decreased, and the oxidation level increased concomitantly with the presence of obesity, the number of MS components, higher coronary risk, and insulin resistance parameters.

**Conclusion:**

MHO presented a similar mtDNA oxidation level to NW and mtDNA content to the MUO, placing the MHO individuals as having an intermediate phenotype. Changes in mtDNA content and oxidation were correlated to the lipid profile related to obesity and/or MS presence, probably associated with oxidative stress and chronic low-grade inflammation.

## 1. Introduction

Chronic noncommunicable diseases (NCDs), such as obesity and associated metabolic diseases such as dyslipidemia, arterial hypertension, diabetes, and cardiovascular complications from their progression, constitute a huge public health problem that, in the last decades, has become a pandemic of uncontrollable proportions. In Argentina, the national surveys of the Ministry of Health showed a 73.28% increase in the prevalence of obesity between 2005 and 2018, reaching 25.4% of the population; actually, approximately 40% of people are overweight, and 26% of people are obese [1].

Obesity with increased visceral or central adiposity entails a higher risk of metabolic and cardiovascular complications than subcutaneous adiposity [2–4]. This localization is crucial for the development of systemic, chronic, and low-grade inflammation that characterizes individuals with obesity. It determines the onset of multiple alterations of metabolic origin such as metabolic syndrome (MS) and is a hazard factor for the development of cardiovascular diseases (CVD) and type 2 diabetes (T2D). The probability of having those complications increases with the number of metabolic risk factors present in an individual [5]. However, a subgroup of obese individuals called metabolically healthy obese (MHO) seems to be protected against the presence of these metabolic alterations, which constitutes the central phenotype of this study [6, 7]. MHO is often considered a transient phenotype, with most individuals transitioning to a metabolically unhealthy status over time [8]. An international consensus of medical societies described MHO individuals as those with a body mass index (BMI) greater than 30 kg/m^2^ and one or less of the five classic MS alterations [9] included in the 15% to 20% of people with obesity who are resilient to developing MS [10]. These individuals have a lower risk of mortality from all causes compared to obese individuals with MS [11], and a meta-analysis recognized that the risk of developing CVD among MHO was significantly lower than in obese individuals [12].

Mitochondrial dysfunction is strongly associated with diseases linked to MS. Mitochondria, the cell's energy-producing centers, are now recognized as central nodes of immune/metabolic modulation that regulate key mechanisms for cell homeostasis. Mitochondria are dynamic, reticular organelles with high plasticity in their structure, forming interconnected networks through continuous fusion and fission processes [13]. The balance between mitochondrial biogenesis, mitophagy, and dynamics directs mitochondrial function, enabling metabolic adaptation to cellular needs. Imbalances in this equilibrium result in morphological abnormalities, leading to inhibition of oxidative phosphorylation, increased production of reactive oxygen species (ROS), and a decrease in FFA *β*-oxidation, contributing to an accumulation of FFAs that favors the development of insulin resistance. The accumulation of FFA is accompanied by an increase in levels of diacylglycerides (DG) and ceramides, which inhibit insulin signaling [14].

Scarce research has been previously carried out with respect to mtDNA content in metabolically healthy obese (MHO) individuals. Adequate clinical, biochemical, and genetic-molecular identification of MHO individuals will allow us to understand in depth the mechanisms that would lead to a lesser progression of cardiovascular disease (CVD) and T2D. Thus, the objectives of our study included the determination of the mtDNA oxidation level and content in different obesity phenotypes (MHO or with metabolic syndrome, MS) while comprehending the associations with diverse clinical, metabolic, and environmental parameters.

## 2. Materials and Methods

### 2.1. Characterization of the Population

The studied population belonged to the city of Venado Tuerto, which is located in the Southwest of the Province of Santa Fe, Argentina. The city is part of the Pampas region, which is the most densely populated, with over 55% of the country's population, and hence representative of the Argentinean population. A total of 286 unrelated individuals of both sexes, aged between 18 and 80 years, were selected. Ninety-seven obese individuals were subclassified as metabolically unhealthy obese (MUO) according to the National Cholesterol Education Program Adult Treatment Panel III (NCEP ATP III) [15], whereas 95 were considered metabolically healthy obese (MHO) following Alberti et al. [9]. The control group included 94 individuals with normal weight (NW) (BMI between 18.5 and 24.9) who had no significant chronic or acute diseases, were not under medical treatment that could affect weight or metabolism, and did not have risk factors for metabolic syndrome.

The individuals were recruited between June 2017 and November 2019. The sampling design was probabilistic, multistage, stratified, and raised by conglomerates of housing units according to the 2010 population and housing census [16]. The individuals were interviewed to collect demographic, familiar, and personal medical history, age, and sex data. Anthropometric measurements, blood pressure, and standardized biochemical studies were performed. The biochemical studies were performed on peripheral blood samples obtained after 12 hours of fasting [16].

Blood pressure was measured with an Omron sphygmomanometer twice (10 minutes apart), and the second measurement was recorded. Height and weight were determined with the subjects dressed in light clothing and without shoes, and the BMI was calculated as weight/(height)^2^ (kg/m^2^). Total cholesterol was measured by the CE/CO/HPO/DEA-HCL/AAP method (Dimension Siemens reagent); high-density lipoprotein cholesterol (HDL-c) and low-density lipoprotein cholesterol (c-LDL) were measured by the homogeneous method (Dimension Siemens reagent); triglycerides were measured by the lipase/glycerol kinase/GPO method (Dimension Siemens reagent). Glycemia was determined by the H method (Siemens Dimension reagent), and glycosylated hemoglobin was determined by high-performance liquid chromatography [17]. Individual's information on smoking, fruit intake, type of sweetener used, consumption of carbonated and alcoholic beverages, type of bread consumed, salt intake, and physical activity was documented. The protocol was approved by the Ministry of Health of the Province of Santa Fe, and the Ethics Committee approved the informed consent, which was signed by all the participants included in the protocol. The researchers followed national (ANMAT Provision 5330/97 and Personal Data Protection Law No. 25, Rachel 6) and international bioethical standards (Declaration of Helsinki in its latest version, Fortaleza 2013, Nuremberg Code and Universal Declaration on the Human Genome and Human Rights).

### 2.2. DNA Purification and Quantification

From each participant, 5 ml of peripheral blood was obtained by venipuncture into Vacutainer tubes containing ethylenediaminetetraacetic acid disodium salt (EDTA) and stored at −20°C until processing. For isolation of peripheral blood leukocytes from blood, 600 *µ*L of whole blood was taken, washed three times with T10E10 buffer (Tris base 10 mM and EDTA 10 mM), and then centrifuged for supernatant removal. For genomic DNA extraction, the pellet was resuspended in CTAB and incubated at 64°C for 2 hours. Subsequently, two extractions with saturated IAC (isoamyl alcohol: chloroform, 24 : 1 v/v) in H_2_O were performed. An equal volume of isopropanol was added, mixed, and precipitated in the freezer overnight. After centrifugation, the supernatant was discarded, and the pellet was air-dried and resuspended in distilled water according to the DNA yield [17]. Finally, DNA purity was determined with *A*_260_/*A*_280_ ratio ∼1.8 and *A*_260_/*A*_230_ ratio ∼2.0 and quantified using spectrophotometry (DeNovix DS-11).

### 2.3. Determination of the mtDNA Oxidation Level (8-Oxoguanine)

We quantified the 8-oxo-guanine residues (8-OxoG) as a product of mtDNA ROS oxidation. Genomic DNA (56 ng) was treated with 0.7 *µ*L of Fpg (8000 U/mL) (DNA-formamidopyrimidine glycosylase, Cat. M0240L™, New England Bio-Labs) for 45 minutes at 37°C, followed by inactivation of the reaction with heat (10 minutes at 60°C). The untreated sample underwent the same procedure but without the addition of the enzyme. The enzyme FPG has N-glycosylase and AP-lyase activity to release purines damaged by oxidation and generate apurinic sites (AP). AP-lyase activity cleaves each AP site through beta and gamma deletion, creating a one-nucleotide DNA gap with 5′ and 3′ terminal phosphate [18]. Consequently, when performing quantitative real-time PCR (qPCR), a delay in the TC (TC: cycle in which the threshold value is exceeded) was observed when compared with the untreated sample, proportionally related to the oxidation level.

The qPCR determinations were made in a final 10-ul reaction volume with 3 ng of DNA, 2.5 nM of SYTO 9, 1.5 mM of magnesium chloride, 0.04 mM of dNTPs, 0.75 U of TaqGo DNA polymerase, and 0.5 *µ*M of primers for the MT-TL1 gene (tRNA-encoded leucine 1 gene). The qPCR primers used were 5′CACCCAAGAACAGGGTTTGT3″ (forward) and 5′TGGCCATGGGTATGTTGTTA3′ (reverse) for the mtDNA amplification. The PCR conditions were as follows: 2 min at 50°C, 20 s at 95°C, followed by 40 cycles of 15 s at 95°C, 20 s at 58°C, and 10 s at 72°C, performed in a thermocycler, Applied Biosystems StepOnev2.3. The melting curve was performed with 1 cycle of 15 s at 95°C and then 20 s of continuous temperature increase between 50°C and 95°C with a ramp of 0.1°C/s. Results were calculated as ∆TC/mtDNA (%), where ∆TC is the difference between the untreated sample and the FPG enzyme-treated sample.

### 2.4. Determination of mtDNA Content

For the determination of mtDNA content, a set-up was performed with interassay variability assessed for two mitochondrial genes: ND1 (mitochondrial gene encoding NADH-ubiquinone oxidoreductase) [19] and MT-TL1; and for two nuclear genes: *β*2M (*β*2 microglobulin) and 36B4 (gene encoding ribosomal acid phosphoprotein) [20]. MT-TL1 and *β*2M genes were selected due to the lower interassay variability observed (CV: 3.21 and 1.40).

Two consecutive PCRq reactions were performed for each DNA sample: the first to amplify an 85-bp fragment of the nuclear *β*2M gene, and the second to amplify a 108-bp fragment of the MT-TL1 mitochondrial sequence. The same primers as before were used for the mtDNA amplification and for *β*2M: 5′TGCTGTCTCCATGTTTGATGTATCT3″ (forward) and 5′ TCTCTGCTCCCCACCTCTAAGT3′ (reverse) [21].

The previously described mtDNA amplification protocol for the determination of the mtDNA oxidation level was used with a primer annealing temperature of 61°C. Each sample was analyzed in duplicate, and all measurements included the determination of the positive control, a reference sample, and a negative control without a template.

Results were calculated using the comparative cycle threshold method. The mtDNA content was calculated using the following formula: 2 x 2^−ΔCT^, where ΔCT is the difference between the CT of the *β*2M gene and the MT-TL1 gene. A control DNA sample was prepared with peripheral blood leukocyte DNA from 3 women of different ages in equal proportions. This control was amplified in duplicate for each primer pair in all assays. The relative mtDNA of each patient was calculated by dividing the mtDNA content of the patient by the mtDNA content of the control sample (in percent) [22].

### 2.5. Statistical Analysis

We estimate the sample size according to a pilot test with 47 NW, 52 MHO, and 52 MUO. These outcomes were considered in the determination of the effect size of 0.255. Using Gpower v3.1.9.7, ANOVA for independent samples, a total sample size of 240 individuals was considered sufficient, assuming an alpha level of *p*: 0.05 and a power of 95%. One-way ANOVA and post hoc Tukey test were used to compare biochemical, clinical, and anthropometric characteristics as well as mtDNA content and its oxidation level between groups. Student's *t*-test for independent samples was used to assess the mtDNA content and its oxidation level for the different variables. Linear regression was used to evaluate the association between mtDNA content and oxidation level with the different biochemical, clinical, and anthropometric variables. In all cases, different parameters related to the consumption of food and beverages and smoking were considered as possible confounding variables. All statistical analyses were performed in SPSS v.25 with a significance level of 0.05.

## 3. Results

### 3.1. Phenotypic and Biochemical Characterization of the Study Population

The anthropometric, clinical, and biochemical characteristics of the studied Venado Tuerto population grouped according to metabolic status are shown in Table 1. NW individuals presented significantly lower BMI, waist circumference, systolic blood pressure (SBP), diastolic blood pressure (DBP), and LDL-c level than MHO individuals. In addition, we observed significant differences in all variables when NW individuals were compared to MUO. It was observed that MHO individuals presented significantly lower TG, glycemia, and blood pressure values and a significantly higher HDL-c level compared to the MUO group; no significant differences were found in anthropometric parameters. These results placed MHO as an intermediate phenotype between NW and MUO.

### 3.2. Mitochondrial DNA Oxidation Level and mtDNA Content

**Figure 1:**
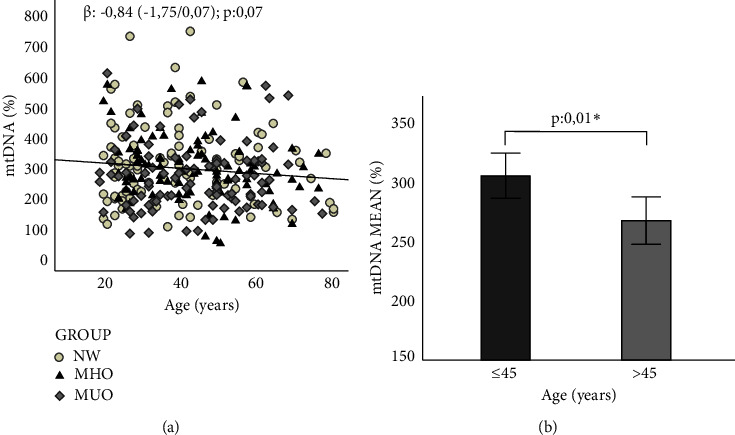
Variation of mtDNA with age. (a) Regression between mtDNA and age in the general population. (b) Comparison of means of mtDNA content according to age. NW: normal weight; MHO: metabolically healthy obese; MUO: metabolically unhealthy obese. Statistical evaluation: linear regression; comparison of means using student's *t* test. ^∗^*p* ≤ 0.05 and ^∗∗^*p* < 0.01 were considered significant.

8-OxoG mtDNA level oxidation and the content of mtDNA were both quantified in peripheral blood leukocytes. A decreasing trend in mtDNA content was observed in the general population as the age of the individuals increased (*β*: −0.84, *p*: 0.07) (Figure 1(a)). We established a cut-off value of 45 years old in which lower mtDNA content was significantly correlated to age for older individuals (305.03 ± 125.80 vs. 267.41 ± 109.03, *p*: 0.01) (Figure 1(b)). No significant age-related changes were observed in the mtDNA oxidation level in all groups (data not shown).

**Figure 2:**
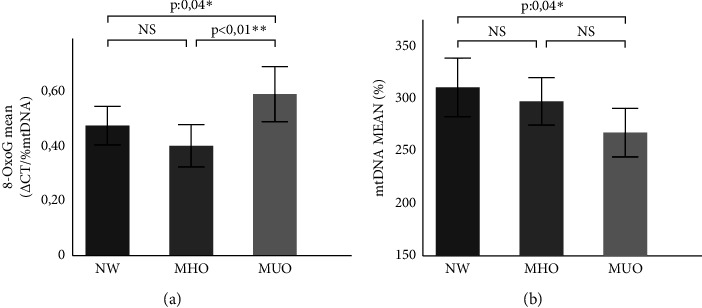
Comparative study of the mtDNA oxidation level and content between NW, MHO, and MUO groups. (a) Comparison of the level of mtDNA oxidation between controls and different obesity phenotypes. (b) Comparison of mtDNA content between controls and different obesity phenotypes. NW: normal weight; MHO: metabolically healthy obese; MUO: metabolically unhealthy obese. Statistical evaluation: Comparison of means using student's *t* test. ^∗^*p* ≤ 0.05 and ^∗∗^*p* < 0.01 were considered significant.

The results of the comparative study among NW, MHO, and MUO groups showed an increase in the 8-OxoG level in MUO compared to the other groups, with significant differences between MUO vs. WHO (0.57 ± 0.49 vs. 0.39 ± 0.36; *p*: 0.05) and NW vs. MUO (0.47 ± 0.33 vs. 0.57 ± 0.49; *p*: 0.04) (Figure 2(a)). In addition, a progressive decrease in mtDNA content was detected among NW, MHO, and MUO individuals (308.59 ± 134.82 vs. 295 ± 110.07 vs. 266.10 ± 113.21), wherever MUO had a significantly lower mtDNA content compared with NW (266.10 ± 113.21 vs. 308.59 ± 134.82; *p*: 0.04) (Figure 2(b)).

**Figure 3:**
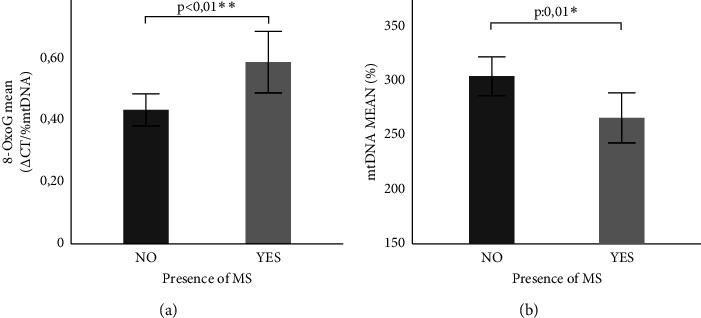
Comparative study of the mtDNA oxidation level and content between the presence and absence of MS. (a) Comparison of the mtDNA oxidation level between the presence or absence of MS. (b) Comparison of mtDNA content between the presence or absence of MS. MS: metabolic syndrome; comparison of means using student's *t* test. ^∗^*p*≤0.05 and ^∗∗^*p* < 0.01 were considered significant.

**Figure 4:**
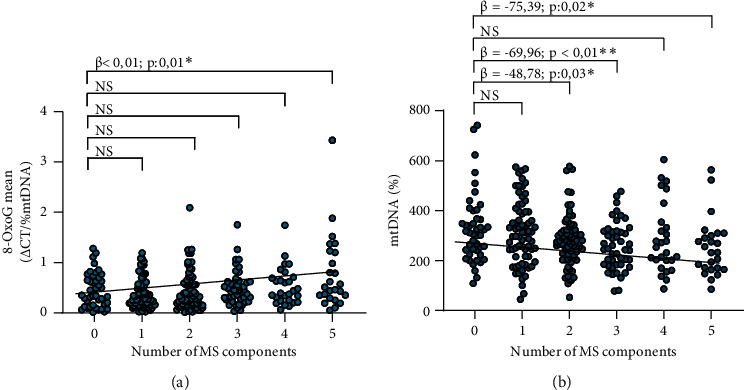
Relationship between the oxidation level of mtDNA and content with the number of MS components (abdominal obesity, elevated triglycerides, low HDL-c, high blood pressure, and elevated fasting glucose) with (a) 8-OxoG and (b) mtDNA content. ^∗^*p*≤0.05 and ^∗∗^*p* < 0.01 were considered significant.

It is noteworthy that an increase in the oxidation level (0.43 ± 0.35 vs. 0.58 ± 0.49; *p* < 0.01) (Figure 3(a)) and a decrease in mtDNA content (302.61 ± 122.31 vs. 264.78 ± 113.40, *p*: 0.01) (Figure 3(b)) were presented in the presence of MS. In this sense, an increase in the mtDNA oxidation level was observed as the number of MS components increased, particularly significant in the presence of all five components (Figure 4(a)). We analyzed the effect of metabolic syndrome components as predictor variables for the mtDNA oxidation level by multiple linear regression, and the model explained 60% of the variation in mtDNA oxidation (*p*: 0.01). All the components were included in the model, observing that glycemia (10.6%) had the greatest effect on mtDNA oxidation, followed by HDL-c levels (8.7%), TG levels (8.3%), diastolic blood pressure (3.5%), waist circumference (1.9%), and systolic blood pressure (1.5%).

Furthermore, a decrease in mtDNA content was observed as the number of MS components increased (Figure 4(b)). Using multiple linear regression, with a model that explains 75% of the variation in mtDNA (*p*: 0.02), we analyzed the effect of metabolic syndrome components as predictor variables for mtDNA content. All the components were included in the model, observing that the variable that has the greatest effect on mtDNA content was HDL-c levels (20.9%), followed by systolic blood pressure (17.6%), TG levels (11.6%), waist circumference (8%), diastolic blood pressure (7.2%), and glycemia (4.1%).

In this sense, the relationship between the mtDNA oxidation level and content was evaluated, considering the lipid profile variables. The mtDNA oxidation level was directly associated with TG levels (*β* < 0.01, *p* < 0, 01) (Supplementary Figure 1A) and inversely related to HDL-c levels (*β*: −0.01, *p*:0.01) (Supplementary Figure 1B). Afterwards, the population was divided using the lipid profile variables and cut-off values, observing a significant increase in the mtDNA oxidation level with a higher TG level (0.44 ± 0.34 vs. 0.59 ± 0.55; *p*: 0.04) (Supplementary Figure 2A) and a lower HDL-c level (0.42 ± 0.33 vs. 0.55 ± 0.48; *p*: 0.01) (Supplementary Figure 2B). We observed a direct correlation of mtDNA oxidation with the blood glucose level (*β* < 0.01; *p*: 0.01) (Supplementary Figure 3A) and glycosylated hemoglobin (*β*: 0.05; *p*: 0.04) (Supplementary Figure 3B).

Furthermore, it was observed that mtDNA content exhibited a positive correlation with HDL-c levels (*β*: 1.70, *p* < 0, 01) (Supplementary Figure 4A) and a negative correlation with LDL-c levels (*β*: −0.47, *p*: 0.05) (Supplementary Figure 4B). Subsequently, the study cohort was stratified based on cut-off values for the analyzed lipid profile variables. Substantial differences were noted for the levels of HDL-c (Supplementary Figure 5A) and LDL-c (Supplementary Figure 5B), concerning their association with mtDNA content. Notably, a reduction in mtDNA content was observed when these lipid parameters were altered (HDL-c 306.27 ± 122.61 vs. 270.77 ± 115.58, *p*: 0.01; LDL-c 301.03 ± 124.86 vs. 271.64 ± 113.68, *p*: 0.05). A downward trend in mtDNA was noticed in correlation with an increasing glycemia level (*β*: −0.35; *p*: 0.10) and glycosylated hemoglobin (*β*: −8.72; *p*: 0.20). Regarding the anthropometric parameters, the means of mtDNA content decreased when IMC (308.02 ± 134.21 vs. 280.62 ± 112.34, *p*: 0.07) and WC (311.51 ± 131.40 vs. 274.23 ± 109.91, *p*: 0.01) cut-off values were considered.

### 3.3. Relationship between the Oxidation Level and mtDNA Content with Cardiovascular Risk

**Figure 5:**
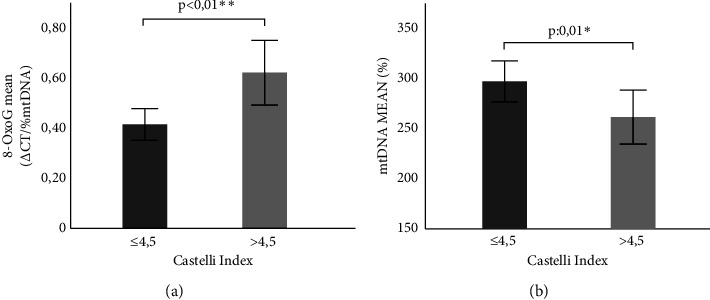
Relationship between the mtDNA oxidation level and content with cardiovascular risk. (a) Comparison of the mtDNA oxidation level according to the Castelli index in obesity. (b) Comparison of mtDNA content according to the Castelli index in obesity. Statistical evaluation: Comparison of means using student's *t* test. ^∗^*p*≤0.05 and ^∗∗^*p* < 0.01 were considered significant.

Considering that the Castelli Index (the ratio between total cholesterol and HDL-c) has a higher predictive risk value for the development of cardiovascular disease than any of the lipid profile variables alone, a significantly lower value was observed in the MHO population compared to the MUO population (3.64 ± 0.84 vs. 5.22 ± 1.61; *p* < 0.01). We evaluated the mtDNA oxidation level and mtDNA content in relation with the Castelli Index, considering a cut-off value of 4.5 [23]. When the cut-off value was exceeded, we observed a greater oxidation level (0.43 ± 0.33 vs. 0.60 ± 0.54; *p*: 0.01) and lower mtDNA content (301.51 ± 122.36 vs. 262.00 ± 115.29; *p*: 0.01) in the general population mainly related to individuals with obesity [Figure 5(a) and 5(b) (0.41 ± 0.34 vs. 0.61 ± 0.55; *p*: 0.01); (295.27 ± 108.39 vs. 259.99 ± 116.35; *p*: 0.03), respectively].

### 3.4. Relationship between the Oxidation Level and mtDNA Content with Insulin Resistance

**Figure 6:**
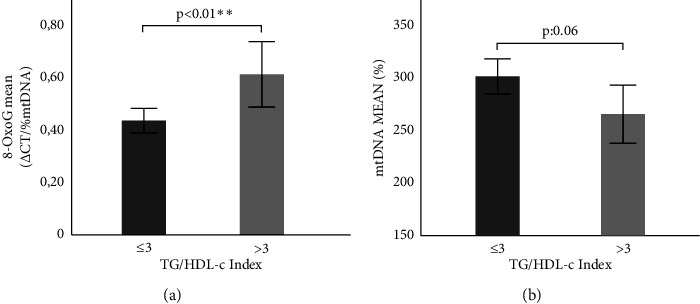
Relationship between the mtDNA oxidation level and content with insulin resistance. (a) Comparison of the mean of the mtDNA oxidation level according to the TG/HDL-c index. (b) Comparison of mean mtDNA content according to the TG/HDL-c index in obesity. Statistical evaluation: Comparison of means using student's *t* test. ^∗^*p*≤0.05 and ^∗∗^*p* < 0.01 were considered significant.

We also decided to analyze the TG/HDL-c index, which could be used as a secondary marker of insulin resistance, given its acceptable sensitivity and specificity [24]. The TG/HDL-c index better identified patients in terms of insulin resistance associated with cardiometabolic risk and higher statistically significant values than variables such as homeostasis model assessment, fasting plasma insulin, blood pressure, body mass index, waist circumference, and fasting glucose levels [25]. It was observed that MHO presents a significantly lower TG/HDL-c index than MUO. (1.88 ± 0.89 vs. 6.08 ± 7.54; *p* < 0, 01). A higher mtDNA oxidation level (0.43 ± 0.33 vs. 0.61 ± 0.55; *p*: 0.01) and lower mtDNA content (300.00 ± 120.57 vs. 264.44 ± 120.95; *p*: 0.03) were observed in the general population mainly due to individuals with obesity, when the cut-off value was exceeded [Figures 6(a) and 6(b) (0.41 ± 0.33 vs. 0.61 ± 0.55; *p*: 0.01) (292.87 ± 106.65 vs. 262.38 ± 120.10; *p*: 0.07), respectively].

## 4. Discussion

Mitochondrial dysfunction has been linked to metabolic syndrome-related diseases. Several studies reported a correlation between the peripheral blood mtDNA copy number and IR, glucose deregulation [26], liver disease [27], hyperlipidemia [28], and various types of cancer [29]. Changes in mtDNA content have been observed in metabolic disorders such as diabetes and obesity [30–32]. In turn, reduction in peripheral blood mtDNA content was found to precede the development of T2D [33]. Wong et al. [34] reported that lower levels of peripheral blood mtDNA were associated with the early development of DBT, but only in those patients without complications. Studies in patients with MS have detected defects in the mitochondrial structure and function and, in turn, high levels of oxidative stress, causing endothelial dysfunction, alterations in metabolism and cellular signaling pathways, protein nitration, lipid peroxidation, and mtDNA damage [35, 36].

In previous works, our group reported that the MHO group would be defined as a subgroup of obese individuals with an intermediate phenotype between NW and MUO individuals, taking into consideration parameters such as HOMA, hs-CRP, and central obesity [37]. Here, we reported that individuals with MS have a higher mtDNA oxidation level and lower mtDNA content compared to NW and MHO individuals. A progressive decrease in mtDNA content was observed between NW, MHO, and MUO individuals. Even though the observed lower mtDNA content in MUO compared to MHO does not reach a significant difference, it was possible to differentiate those groups when mtDNA oxidation level analysis was performed.

Interestingly, no differences were observed in mtDNA oxidation levels between MHO and NW individuals. This could be due to the conservation of mitochondrial homeostasis in both groups, where no significant differences in metabolic variables were observed. It is possible that the mechanisms of mitochondrial repair and turnover are upregulated in MHO individuals, which maintains low levels of ROS and minimizes oxidative damage to proteins, lipids, and DNA. In this way, mitochondrial content and function could be preserved in MHO individuals in a particular obesogenic environment. In summary, MHO exhibited mtDNA oxidation levels similar to those of NW and mtDNA content comparable to that of MUO individuals. These results place MHO as an intermediate phenotype, in agreement with our previous results [37, 38].

Also, we observed a higher mtDNA oxidation level and lower mtDNA content in association with the presence of MS. These results are in agreement with the Fazzini et al. [30] study, which noted a decreased number of mtDNA copies with the increasing number of MS components in the general population. Furthermore, the same study reported that individuals with lower mtDNA content had significantly higher odds of developing MS and T2D.

It must be considered that excess caloric intake and a sedentary lifestyle lead to dyslipidemia with an increase in free fatty acids (FFA), TG, and LDL-c level, which in turn would promote mitochondrial dysfunction. Increased ROS production causes oxidative damage to lipids, proteins, and DNA, activating the fission and mitophagy processes and inhibiting mitochondrial biogenesis, which could explain the high levels of mtDNA oxidation and the decrease in mtDNA content observed in peripheral blood in the MUO group. On the other hand, Casuso and Huertas [39] demonstrated that when leukocytes differentiate into a proinflammatory phenotype, the mitochondrial network is fissioned and mitophagy increases. Based on this observation, we can explain that mtDNA content in peripheral blood leukocytes would be decreased in environments of chronic low-grade inflammation, such as those observed in individuals with obesity and/or MS.

In turn, the current study supports previous observations related to a decline of the mtDNA copy number with age [40, 41]. We have reinforced these findings by demonstrating that the progressive decrease in mtDNA content seems to start in middle age, at approximately 45 years of age. Similarly, Mengel-From et al. [42] observed that the number of mtDNA copies in peripheral blood cells was similar in people aged 18 to 48 years, with a decrease after 50 years.

In this work, we determined that mtDNA content correlated with MS components such as HDL-c and WC. Although LDL-c is not considered among the risk variables for MS, the correlation could be explained by LDL-c oxidation and its low affinity for the receptor, with the consequent increased values at the peripheral level. More studies are required to determine the extent of the observed results. The augmented prevalence and risk of MS could be attributed to the loss of mitochondrial homeostasis through increased oxidative stress and inflammation compared with MHO individuals. Currently, measurements are being made to assess oxidative stress and inflammation levels in our group of patients. Other mitochondrial parameters studied such as function, shape, dynamics, biogenesis, mitophagy, lipid peroxidation, and genetic and epigenetic aspects will contribute to the characterization of the studied groups.

It must be considered that the correlation between the mtDNA oxidation state and mtDNA content of leukocytes with the levels of HDL-c and LDL-c observed in the present study underlines their impact in MS and is consistent with previous studies [18]. In this way, the high cardiovascular risk denoted by a Castelli Index value greater than 4.5 and the higher insulin resistance denoted by a TG/HDL-c Index value greater than 3 were related to an increased oxidation level and decreased mtDNA content.

Although mitochondrial bioenergetics are influenced by genetic, environmental, and lifestyle factors, cellular mitochondrial content positively correlates with proper cell function and metabolism. While the mtDNA content within each mitochondrion is dynamic, its measurement and oxidation state serve as good indicators of mitochondrial dysfunction due to the oxidative stress characteristic of metabolic states such as obesity and metabolic syndrome. The MHO individuals selected in this work do not exhibit insulin resistance, consistent with an increase in mtDNA levels and a decrease in oxidation levels observed when comparing these individuals with those presenting MS and IR. The inverse and direct relationship found between mtDNA content and its oxidation level, respectively, with insulin resistance determined by TG/HDL-c reinforces the hypothesis that mtDNA content reflects mitochondrial cellular content and function, and a decrease in its concentration may precede obesity and IR. These results contributed to the characterization of MHO individuals as having an intermediate and favorable phenotype in terms of their lipid profile, cardiovascular risk, and insulin sensitivity.

In conclusion, based on our results, MHO individuals are classified as having an intermediate phenotype between individuals with NW and MUO, which supports previous studies that describe MHO individuals as those with a lower risk of mortality from all causes compared to obese individuals who develop MS [11]. Changes in mtDNA oxidation and mtDNA content could be explained by oxidative damage caused by oxidative stress and inflammation associated with obesity and/or MS.

Medical consensus and current guidelines for the treatment and prevention of obesity complications do not distinguish between MHO patients and those with MS. The MHO phenotype should be considered a multifactorial entity which deserves to be identified and better characterized, and no animal models are adequate to define the influential factors involved in the possibility or not of developing MS.

The biochemical, molecular, and genetic studies of MHO patients could have a favorable impact in the clinical practice and in public health since they would identify new molecular targets with the possibility of intervention to delay, attenuate, or prevent the development of obesity-related complications in obese people with a higher metabolic risk. Our study focused on the characterization of mtDNA in terms of its contribution to obesity phenotypes. Even though peripheral blood leukocytes are a heterogeneous cell population, the lack of studies characterizing mitochondrial homeostasis in tissues affected by MS and its correlation in peripheral blood makes leukocyte mtDNA content and oxidation potential tools in the prediction of changes in systemic metabolic function [43]. The results of this study support the hypothesis that a minimally invasive method, such as the determination of mtDNA content and its oxidation level in human peripheral blood, could serve as an indicator of numerous metabolic abnormalities. Its relevance as an early predictor of MS development is particularly noteworthy.

## 5. Conclusions

As far as our current knowledge extends, this is the first report determining the level of mtDNA oxidation in the different obesity phenotypes. We characterized MHO individuals as having an intermediate phenotype in terms of mtDNA content and its oxidation level between NW and MUO groups. Both parameters are detectable in peripheral blood leukocytes and could be considered as biomarkers of metabolic and anthropometric changes in metabolic diseases. Moreover, they are complementary to the clinical and biochemical parameters that contribute to the characterization of insulin resistance and cardiovascular risk in MHO individuals.

## Figures and Tables

**Table 1:** Anthropometric, clinical, and biochemical characteristics of the Venado Tuerto population grouped according to metabolic status.

Features	NW (*n* = 94)	MHO (*n* = 95)	MUO (*n* = 97)	P	P NW vs. MHO	P MHO vs. MUO	P NW vs. MUO
Age (years)	39.5 ± 16.75	43.04 ± 14.44	43.70 ± 14.65	NS	NS	NS	NS
WC (cm)	77.06 ± 6.99	103.46 ± 11.32	110.14 ± 10.63	<0.01^b^	<0.01^b^	<0.01^b^	<0.01^b^
BMI (kg m^−2^)	22.28 ± 1.62	34.99 ± 5.25	35.50 ± 5.09	<0.01^b^	<0.01^b^	NS	<0.01^b^
Glycemia (mg dl^−1^)	94.20 ± 13.94	95.38 ± 9.63	124.22 ± 49.97	<0.01^b^	NS	<0.01^b^	<0.01^b^
Glycosylated hemoglobin (%)	5.31 ± 0.48	5.47 ± 0.45	6.16 ± 1.58	<0.01^b^	NS	<0.01^b^	<0.01b
TC (mg dl^−1^)	186.64 ± 39.87	199.94 ± 34.72	203.58 ± 40.44	0.01^a^	0.05^a^	NS	0.01^a^
TG (mg dl^−1^)	83.64 ± 40.1	100.37 ± 35.07	208.76 ± 155.63	<0.01^b^	NS	<0.01^b^	<0.01^b^
HDL-c (mg dl^−1^)	58.81 ± 14.16	57.05 ± 13.20	41.27 ± 10.24	<0.01^b^	NS	<0.01^b^	<0.01^b^
LDL-c (mg dl^−1^)	108.78 ± 29.83	119.71 ± 27.94	125.43 ± 30.36	<0.01^b^	0.04^a^	NS	<0.01^b^
SBP (mmHg)	116.19 ± 16.34	123.81 ± 20.83	137.37 ± 19.06	<0.01^b^	0.02^a^	<0.01^b^	<0.01^b^
DBP (mmHg)	74.23 ± 10.5	78.70 ± 15.09	89.38 ± 12.34	<0.01^b^	0.05^a^	<0.01^b^	<0.01^b^

Values are expressed as mean ± SD (ANOVA and Tukey post hoc). NW: normal weight group, MHO: metabolically healthy obese, MUO: metabolically unhealthy obese, WC: waist circumference, BMI: body mass index, TC: total cholesterol, TG: triglycerides, HDL-c: high-density cholesterol, LDL-c: low-density cholesterol, SBP: systolic blood pressure, DBP: diastolic blood pressure. a: *p* ≤ 0.05 was considered significant, b: *p* < 0.01 was considered significant.

## Data Availability

Some or all datasets generated during and/or analyzed during the current study are not publicly available but are available from the corresponding author upon reasonable request.

## Supplementary Materials

Supplementary Materials The authors provide supplementary materials which contain figures to complement the results obtained regarding the oxidation level and mtDNA content in relation to their association with the lipid profile and glucose levels. Supplementary Figure 1: mtDNA oxidation level and its relationship with the lipid profile. Regression between 8-OxoG and TG, as well as between 8-OxoG and HDL-c. Supplementary Figure 2: mtDNA oxidation level and its relationship with the lipid profile. Comparison of means of the mtDNA oxidation level considering the cut-off values for TG and HDL-c. Supplementary Figure 3: Relationship between mtDNA oxidation and glycemia. Regression between 8-OxoG and glycemia, as well as between 8-OxoG and glycosylated hemoglobin. Supplementary Figure 4: mtDNA content and its relationship with the lipid profile. Regression between mtDNA and HDL-c, as well as between mtDNA content and LDL-c. Supplementary Figure 5: mtDNA content and its relationship with the lipid profile. Comparison of means of mtDNA content considering the cut-off values for HDL-c and LDL-c.
