# Supplementary material for: Relationship of Mitochondrial DNA Oxidation and Content with Metabolic Syndrome and Cardiovascular Risk in Obesity Phenotypes
Source: J Obes. 2024 Sep 11;2024:3008093. doi: 10.1155/2024/3008093 (PMC11410407; doi:10.1155/2024/3008093)

Supplementary Fig. 1

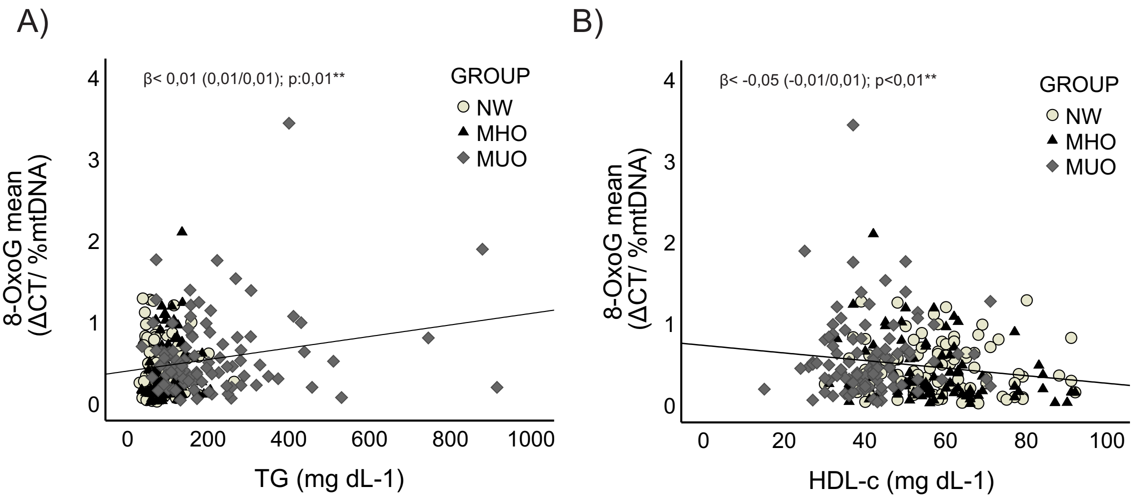

Supplementary Fig. 2

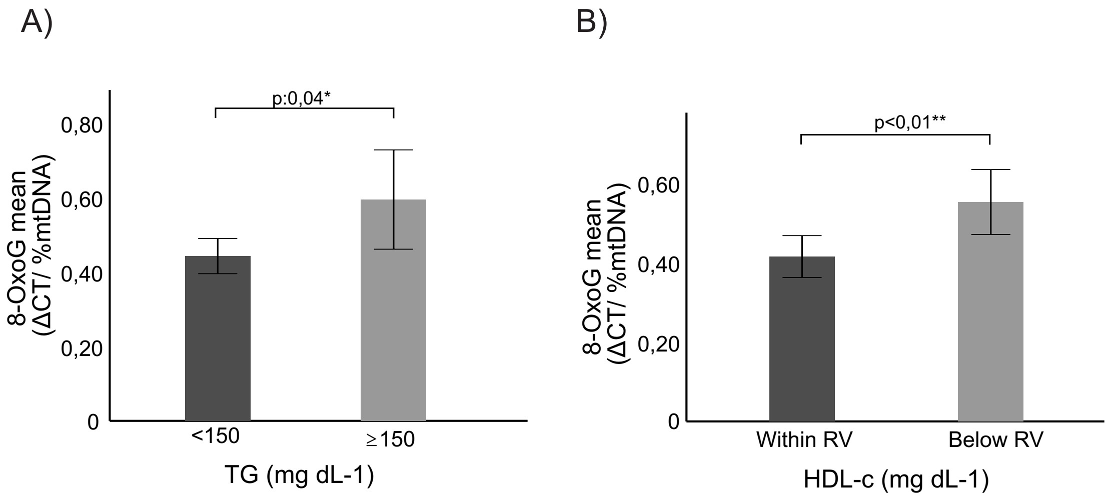

Supplementary Fig. 3

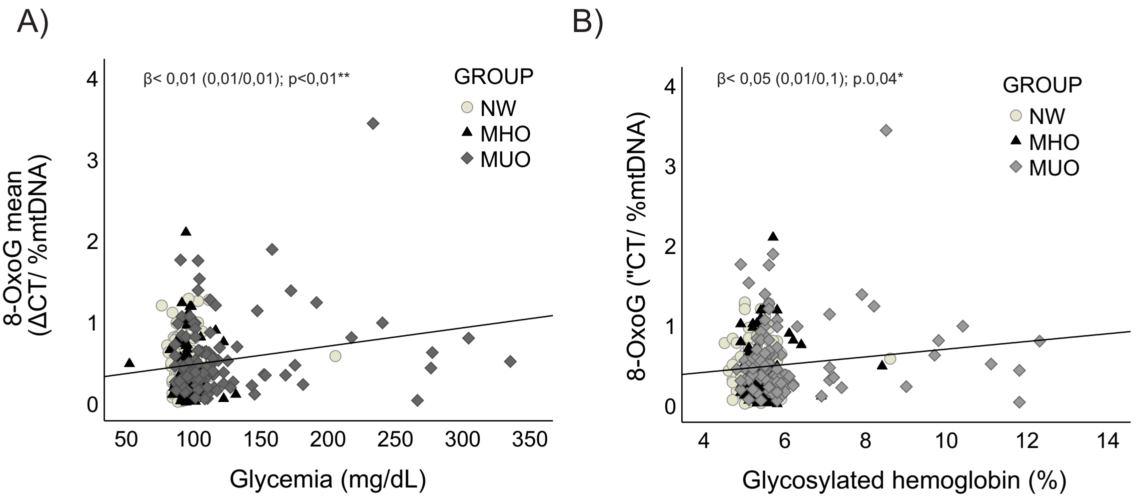

Supplementary Fig. 4

A)

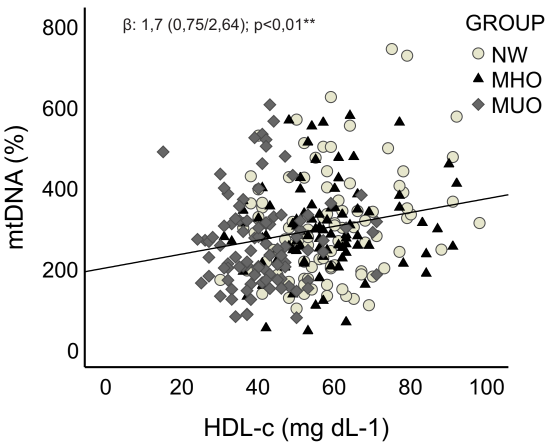

B)

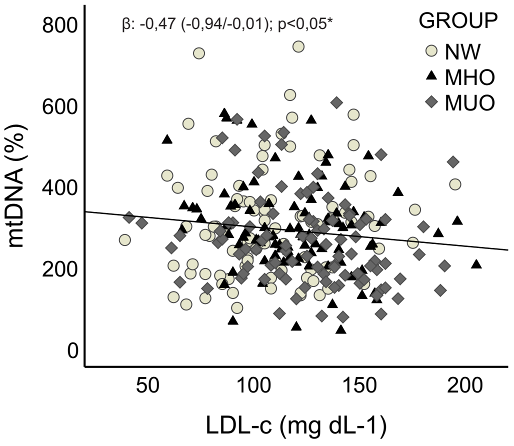

Supplementary Fig. 5

A)

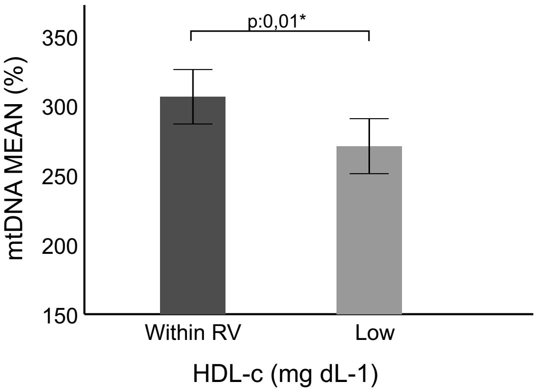

B)

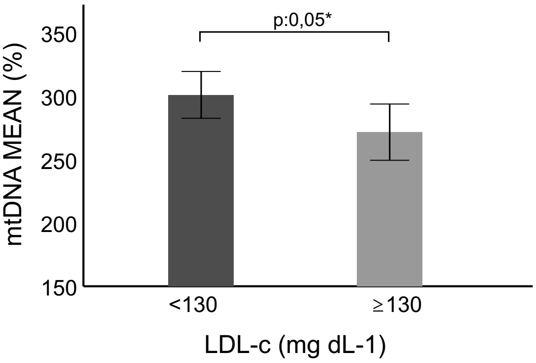

Supplement: Supplementary Materials — The authors provide supplementary materials which contain figures to complement the results obtained regarding the oxidation level and mtDNA content in relation to their association with the lipid profile and glucose levels. Supplementary Figure 1: mtDNA oxidation level and its relationship with the lipid profile. Regression between 8-OxoG and TG, as well as between 8-OxoG and HDL-c. Supplementary Figure 2: mtDNA oxidation level and its relationship with the lipid profile. Comparison of means of the mtDNA oxidation level considering the cut-off values for TG and HDL-c. Supplementary Figure 3: Relationship between mtDNA oxidation and glycemia. Regression between 8-OxoG and glycemia, as well as between 8-OxoG and glycosylated hemoglobin. Supplementary Figure 4: mtDNA content and its relationship with the lipid profile. Regression between mtDNA and HDL-c, as well as between mtDNA content and LDL-c. Supplementary Figure 5: mtDNA content and its relationship with the lipid profile. Comparison of means of mtDNA content considering the cut-off values for HDL-c and LDL-c. [file 3008093.f1.pdf]
